# Maternal and health care workers’ perspectives on exclusive breastfeeding in the context of maternal HIV infection, in Busia county, western Kenya: a mixed methods cross-sectional survey

**DOI:** 10.1186/s13006-022-00454-z

**Published:** 2022-03-04

**Authors:** Esther Clyde Nabakwe, Omar Egesah, Grace Adisa Kiverenge-Ettyang

**Affiliations:** 1 Department of Child Health and Pediatrics, School of Medicine, College of Health Sciences, Moi University, P.O. Box 4606-30100, Eldoret, Kenya; 2 Department of Anthropology, School of Arts and Social Sciences, Moi University, Eldoret, Kenya; 3 Department of Human Nutrition, School of Public Health, College of Health Sciences, Moi University, Eldoret, Kenya

**Keywords:** Exclusive breastfeeding, HIV-infected mothers, Health education, Nutritional counselling, Nutritional supplementation, Mentor mothers, Live case demonstrations

## Abstract

**Background:**

World Health Organization recommends exclusive breastfeeding (EBF) for 6 months with maternal active antiretroviral therapy (ART) to prevent mother-to-child transmission (PMTCT) of HIV. However, EBF in low resource settings remains low. We explored perspectives of EBF by HIV-infected mothers and health care workers in Busia County with a high prevalence of HIV to understand factors influencing the practice.

**Methods:**

A mixed methods cross-sectional survey using concurrent quantitative and qualitative data collection methods was conducted at PMTCT clinics. Data on socio-demography, young infant feeding practices, maternal and infant health was collected between February 2013 and August 2015 from 371 purposively sampled HIV-infected mother-infant dyads using a semi-structured questionnaire. Focus group discussions with mothers, in-depth interviews and passive observation of health care workers during interaction with mothers were conducted. Significance of difference between mothers practicing EBF or not was tested by Chi-square and Fisher’s exact tests setting significance level at 5%. Qualitative data was coded and content analyzed to generate themes.

**Results:**

Three hundred and forty-nine (94%) mothers practiced EBF. Maternal comprehension of EBF to PMTCT of HIV influenced choice and practice of EBF (*P* value = 0.019 and < 0.001 respectively). Health care workers emphasized adherence to ART and offered nutritional supplementation during EBF. Health care workers’ nutritional counseling in the context of maternal HIV was poor. Mentor mothers shared their experiences with mothers and offered live case demonstrations of their successfully EBF, healthy and HIV-uninfected children. The main threats to EBF were teenage motherhood, low maternal education and working during EBF.

**Conclusions:**

EBF among HIV-infected mothers in Busia County, Kenya was high. Health education and counselling by health care workers, maternal comprehension of ART adherence to PMTCT of HIV, nutritional supplementation and mentor mothers’ peer counseling using live case demonstrations of HIV-uninfected EBF children promoted and sustained practice of EBF for 6 months. Teenage motherhood, low maternal education and having to work threatened EBF,

## Background

Breastfeeding is beneficial to both the infant and mother. Worldwide, lactation prevents 13% of infant mortality [1]. Prolonged breastfeeding reduces the risk of obesity, diabetes mellitus and metabolic syndrome due to constituent hormones and growth factors [2–9]. UNAIDS Global Plan towards elimination of new HIV infections among children by 2015 and keeping their mothers alive aimed to promote and support breastfeeding and the provision of lifelong antiretroviral treatment as the strategy to optimize HIV-free survival among HIV-exposed infants, uninfected infants and children [10]. Exclusive breastfeeding (EBF) is feeding the infant on breast milk only without additional feeds or water except medicines, vitamins and mineral supplements. Exclusive replacement feeding (ERF) is feeding the infant only on other foods such as infant formula if acceptable, feasible, affordable, safe and sustainable.

In 2016, an additional 740,000 women of reproductive age world-wide became HIV positive, 73% of whom lived in 23 prevention of mother-to-child transmission (PMTCT) priority countries, majority of which are in sub-Saharan Africa. Kenya is listed as one of these countries. Eighty seven percent of all children and adolescents living with HIV in the world reside here; 87% of new HIV infections among children and 81% of new HIV infections in adolescent girls and young women aged 10–24 years occurred here [10]. Mother-to-child transmission (MTCT) of HIV could occur during pregnancy, delivery or breastfeeding. Without antiretroviral drugs (ARVs), 15–45% of infants born to HIV-infected mothers get HIV infection [11] while with ARVs the rate of transmission is reduced to less than 5% among breastfed infants and less than 2% among formula fed infants [12–14]. This evidence suggest that in order to eradicate HIV, preventive strategies should target women of child bearing age and their infants in the 23 PMTCT priority countries. PMTCT of HIV through breastfeeding is one of these strategies.

Since 2006 there have been several changes in infant feeding and ARVs use in the context of maternal HIV infection [15–17] as shown in Table 1. In 2010, WHO recommended for the first time ARVs interventions to PMTCT of HIV through breastfeeding. WHO consolidated guidelines were updated in 2013 and again in 2016 according to which HIV-infected mothers supported to adhere to antiretroviral therapy (ART) should EBF their infants for the first six months of life, then introduce appropriate complementary foods while continuing to breastfeed for at least twelve months and upto 24 months or longer. When ARVs are not immediately available, mothers should exclusively breastfeed for the first 6 months of the infant’s life and continue unless environmental and social circumstances are safe for and supportive of ERF [18]. However, data on HIV testing and tripple ART coverage in Global Plan priority countries is limited [10]. A systematic review of studies conducted in East Africa revealed a pooled prevalence of MTCT of 7.68% which is more than the desired target of WHO of less than 5% in breastfeeding populations. The authors concluded that strengthening the prevention of vertical HIV transmission, promotion of EBF, timely initiation of ART prophylaxis for HIV exposed infants, encouragement of hospital delivery, and the start of ART at the tme of diagnosis of every HIV-positive person may all reduce the chance of vertical HIV infection [19].

**Table 1:** Antiretroviral Drug Use and infant feeding in the context of maternal HIV infection

Year	Mother receives			Infant receives
**2006**	Treatment for CD4 count < / = 200 cells/mm^3^	Treatment for CD4 count > 200 cells/mm^3^		
	Lifelong ART AZT + 3TC + NVP	ARV prophylaxis starting at 28 weeks of pregnancy AZT twice daily, single dose NVP at onset of labor, AZT + 3TC during delivery and 1 week postpartum		Prophylactic ARVs for 1 week
**2010**	Option	Treatment for CD4 count < / = 350 cells/mm^3^	Prophylaxis for CD4 count > 350 cells/mm^3^	
	A	Triple ARVs starting as soon as diagnosed and continued for life	Antepartum: AZT from 14 weeks, intra-partum at onset of labor sd NVP and first dose AZT/3TC Post-partum: daily AZT/3TC through 7 days post-partum	Daily NVP from birth through 1 week beyond complete cessation of BF (if not BF or mother on treatment through ages 4–6 weeks)
		Same initial ARVs for both		
	B	Triple ARVs starting as soon as diagnosed & continued for life	Triple ARVs from 14 weeks continued intra-partum and through child birth if not breastfeeding (until 1 week after cessation of BF)	Daily NVP or AZT through 4–6 weeks regardless of feeding method
		Same for treatment and prophylaxis		
	B +	Triple ARVs regardless of CD4 count starting as soon as diagnosed and continued for life		Daily NVP or AZT through 4–6/52 regardless of feeding method

*AZT* zidovudine, *3TC* lamivudine, *NVP* nevirapine, *sdNVP* single dose nevirapine, *BF* breastfeeding

In 2012 Kenya adopted option B plus strategy to prevent MTCT of HIV according to which soon after the pregnant women’s HIV status is confirmed, a life-long triple ART is offered regardless of the CD4 count and clinical stage of HIV infection [20]. According to the second Kenya AIDS Indicator Survey of 2012, 6.1% of pregnant women were HIV-infected and less than 75% of these received ARVs [21]. A retrospective review of records of HIV-exposed HIV polymerase chain reaction (PCR) results in Nairobi County, Kenya in 2015 revealed that highly active ART was given to 60.3% of mothers of HIV-exposed infants 5.9% of whom tested positive; 53% of the HIV positive infants received active ART [22]. In the South Rift region of Kenya, a retrospective analysis of PCR results of HIV-exposed infants indicated that mixed feeding was associated with increased HIV transmission. Among these infants, 67.3% were EBF, 10.5% were replacement fed while 8.1% were mixed fed [23].

In Zambia, rapidly changing international guidelines on young infant feeding confused mothers and nurses [24]. In the absence of updated young infant nutritional counseling information by health workers, the series of changes and varied senders of messages on young infant feeding will result into conflicting information passed over to HIV-infected mothers thus leaving them in a confused state and dilemma as to which practice is the best for the survival of their infants. Mothers are torn in between normative cultural practice and medical knowledge messages, information and practice required to appropriately feed their young infants. Information targeting HIV-infected mothers might spill over to HIV negative mothers and vice versa thus negating efforts that have been made over the last two decades to promote and support EBF while reducing MTCT of HIV.

Maternal and health care workers perspectives on EBF during the first 6 months in the context of maternal HIV infection in Busia County of westwern Kenya under rapidly changing WHO guidelines on young infant feeding are not known. This study was designed to explore maternal and health care workers’ perspectives of EBF during the first 6 months. Understanding perspectives of EBF by HIV-infected mothers and health care workers will guide health care providers and policy makers in designing promotion and supportive strategies of EBF incorporating experiences attached to the practice by the main stakeholders – breastfeeding mothers and health care workers.

## Methodology

### Setting

The study was conducted in Busia County, western Kenya where infants are at high risk of MTCT of HIV due to higher prevalence of HIV estimated at approximately 10%, compared with the national average of about 7% [25]. The setting was PMTCT of HIV clinics in 3 hospitals and 7 health centers supported by United States Urgency for International Development and Academic Model Providing Access to Health Care Project. An average of 8–10 and 5–7 mother infant-dyads among other patients are seen at each of the hospitals and health centers respectively. All pregnant and breastfeeding mothers receive active ART regardless of the CD4 count. Breastfeeding infants are given prophylactic ARVs to prevent MTCT of HIV. Monitoring of infant growth and adherence to active ART is done monthly. Nutritional support to the pregnant and breastfeeding HIV-infected mothers is in the form of vitamins, hematinic and 9 kg monthly rations of corn soy blend flour for porridge which is supplied by World Food Program. Psychosocial support is offered by health care workers and peer social support groups where mothers disclose their HIV status and share experiences.

### Design

A mixed methods cross-sectional survey applying concurrent qualitative and quantitative data collection methods was conducted from February 2013 to August 2015.

### Objective

To explore maternal and health care workers’ perspectives on EBF during the first 6 months in the context of maternal HIV infection in order to understand factors positively and negatively influencing the practice.

### Target population

Mothers with infants aged 6 weeks to 6 months and health care workers at PMTCT of HIV clinics in Busia, Kenya.

Inclusion Criteria:

1. Mothers aged 15 -45 years attending PMTCT of HIV clinics with 6 weeks with to 6-month-old infants who consented to participate in the study.
2. Health care workers offering services at PMTCT of HIV clinics for at least 3 months before the study period.

Exclusion Criteria:

1. Non-Kenyan citizenship
2. Refusal to consent
3. Congenital anomalies of infants
4. Health care workers offering services at the clinics for less than 3 months before the study

### Samples size

Fischer’s formula was used to estimate the minimum sample size of mothers required for the questionnaire based on the prevalence of EBF among HIV-infected mothers of 35% in Kitale district hospital, western Kenya [26]. This is because there was no data on exclusive breastfeeding among HIV-infected mothers in Busia County and that Kitale District hospital was the nearest hospital where a study on infant feeding practices among HIV infected women receiving PMTC services had been conducted. A minimum sample of 349 mother-infant dyads would allow estimation of the proportion of mothers who EBF with a precision of 5%.

### Sampling

USAID-AMPATH-Plus Project facilities which included 1 county hospital, 2 sub-county hospitals and 7 health centers were the sampling frame from which HIV-infected mothers were sampled. Three hundred and seventy-one 15–45 year old mothers with 6 month old infants attending PMTCT clinics were sampled purposively by the principal investigator and clinical officers at PMTCT clinics and consecutively recruited in the study to enable complete data set for analysis. Thirty two health care workers at PMTCT of HIV clinics for at least 3 months during the study period were purposively sampled for in-depth interview. Eight focus group discussions (FGDs) were conducted till the point of saturation when no new information emanated from the discussions.

### Data collection tools

A questionnaire was used in order to maintain consistency of the questions posed to the mothers and hence validity of the responses obtained. Focus group discussions (FGDs) were used to identify the experiences and perspectives of the mothers on factors influencing EBF. They aided to uncover unique perspectives on EBF due to the group environment in which data are collected [27]. FGDs prompted members to give more detailed account of issues influencing EBF among them and other mothers in the community. In-depth interviews were used to get the rare experiences of mothers practicing EBF during interaction with the health workers [27].

### Questionnaires

A questionnaire developed by the researcher in English was translated into Kiswahili – a language which subjects in the setting understood. This was back translated into English by a second person experienced in translation of Kiswahili to English in order to confirm consistency of the meaning. This was validated by pilot testing on a sample of 30 HIV-infected mothers with infants not more than 6 months of age. This facilitated necessary corrections to ensure that the subjects understood the questions in order to ensure consistency of the responses and hence reliability of the data generated.

Interviewer-administered structured questionnaires with closed and open ended questions were used to collect quantitative and qualitative data from 371 mothers on demographic characteristics and advice on EBF during pregnancy and postnatal periods till infants attained the age of 6 months. The infant’s exact age was derived from the birthday indicated in the well-baby clinic booklet. Using maternal recall, information on morbidity from common infant illnesses such as upper respiratory tract infections, pneumonia, diarrhea and malaria since birth was collected to determine the health status of the infants during the first 6 months of life based on the feeding option. FGDs with the mothers, in-depth interviews and passive observations of health care workers were conducted to triangulate this data.

### Focus group discussions

Mothers with 6 weeks-6 months old infants not involved in the survey using the questionnaire were eligible for FGDs. They were booked to come to the clinic on a convenient date when at least 8–12 mothers would be available for FGD. Eight FGDs using a topic guide were conducted at PMTCT of HIV clinics to understand the perspectives of mothers on EBF. The focus of the FGD was to establish whether HIV-infected mothers understood the meaning of EBF; which mothers were likely or unlikely to practice EBF and why; factors that influence EBF, support received to ensure that their infants were EBF and the influence of health care workers on choice of infant feeding. This was conducted by the researcher and an assistant moderator for 1 -1·5 h using a pretested FGD topic guide. The FGD were conducted and audio recorded at a private spot away from other mothers and health care workers in order to understand one another and for assurance of confidentiality.

### In-depth interviews

Thirty two health care workers at PMTCT of HIV clinics were interviewed and audio recorded for 1–1·5 h by the principal investigator and an assistant moderator using an interview guide on knowledge and perspectives of EBF in the context of maternal HIV infection. In-depth interview guide focused on the experience of health care workers during their interaction with HIV-infected mothers with infants from birth till the age of 6 months; which mothers are most or least likely to practice exclusive breastfeeding; which factors have positive and negative influence on EBF; what support was offered to ensure that most infants were fed according to the latest WHO recommendation; how health workers influence the choice of feeding babies; what they would recommend to promote, support and sustain exclusive breastfeeding. The interviews were continued until no new themes emerged from the data.

### Observations

Thirty two health care workers were observed during their interaction with the mothers by passive participation of the researcher. The aim was to assess their practice and accuracy of counselling information on infant feeding given to HIV-infected mothers and find out whether they perform nutritional counseling or not. Their knowledge and skills on young infant feeding counseling were assessed and scored against a check list by the researcher using a counseling flow chart derived from HIV and infant feeding counseling tools based on United Nations policies and guidelines [28]. These state that all HIV infected mothers should receive counseling which includes provision of general information about the risks and benefits of various infant feeding options and specific guidance in selecting the option most likely to be suitable for their situation. Whatever the mother decides she should be supported in her choice. The aspects that were observed were 15 in total. The score was carried out after the health care workers had interacted with several mothers and forgot that they were being watched. Each aspect of knowledge and skill was assigned a score of 1 and a percentage out of a total score of 15 computed. A score less than 50% was rated poor, 51 – 64% pass, 65 – 74% credit and 75 – 100% distinction.

### Ethical considerations

The National Council of Science and Technology through Moi University and Moi Teaching and Referral Hospital Research and Ethics Committee approved the study – Formal Approval number: FAN: IREC 000,916. Permission was sought from the Director of USAID-AMPATH-Plus Project and the Medical Officer of Health of Busia County. Informed written consent was sought from health care workers and mothers. Principles of confidentiality, respect for persons, beneficence and justice were adhered to during the study period. All mother-infant dyads received standard health care according to WHO and USAID-AMPATH-Plus Project guidelines.

### Data management and analysis

Quantitative data was cleaned, coded and entered in IBM SPSS version 20 for storage and analysis by the principal investigator with the assistance of a biostatistician. Descriptive statistics were used to summarize the data. Chi-square test and Fisher’s Exact tests were used to determine the differences between infants and mothers subjected to EBF or not. A 5% level of statistical significance was used. Qualitative data was audio recorded, transcribed, labeled and content analysis done to generate themes.

## Results

### Socio-demographic characteristics

Median maternal age was 30 (IQR: 25–33). Most mothers were between 25–35 years of age, married, achieved primary level education and unemployed. Two hundred (54%) of the infants were males. Thirty two health workers were observed and 26 interviewed; 25 (78%) were females. These were Kenya Enrolled Community Nurse, Kenya Registered Community Health Nurse, Nursing Officer, Nutritionist and Clinical Officer (Table 2). Most health care workers were clinical officers. Criteria for selection of mentor mothers were: HIV-infected mothers with healthy HIV-negative children at 18 months of age, strict adherence to ART, successful EBF for 6 months, and good communication skills.

**Table 2:** Maternal and Health Care Workers’ Socio-demographic Characteristics

Subjects	Characteristic	Detail	Number (%)
**Mother (*n* = 371)**	**Maternal age (years)**	< 25	79(29·3)
		25–35	243(65·5)
		> 35	49(13·2)
	**Marital status**	Married	310(83·6)
		Single	32(8·6)
		Separated	19(5·1)
		Divorced	5(1·3)
		Widowed	5(1·3)
	**Education**	None	10(2·7)
		Primary	280(75·5)
		Secondary	81(21·8)
	**Occupation**	Unemployed (business)	255(68·7)
		Informal employment	62(16·7)
		Peasant farmer & business	26(7)
		Casual laborer	12(3·2)
		Formal employment	8(2·2)
			8(2·2)
**Health care worker (*n* = 32)**	**Age (years**	20- < 30	4(12)
		40- < 50	22(69)
		50- < 60	5(16)
			1(30
	**Gender**	Male	7(22)
		Female	25(78)
	**Cadre**	Mentor mothers	4(12)
		KECN^a^	3(9)
		Nutritionist	5(16)
		Nursing Officer	1(3)
		Clinical officer	12(38)
		Psychosocial worker	1(3)
		KRCHN^b^	6(19)

^a^Kenya Enrolled Community Nurse

^b^Kenya Registered Community Health Nurse

Distribution of mothers by health facility is shown in Table 3. Ninety two (25%) of the mothers were in Mukhobola, 90 (24%) in Khunyangu, 82 (22%) in Busia County hospital and 50 (14%) in Port Victoria 37(10%) in Teso sub-county hospital.

**Table 3:** Maternal Distribution by Health Facility & Clinic of Counseling on Exclusive Breastfeeding

	**Facility**	**Frequency (%)**
**Health Facility**	**Angurai**	4(1·1)
	**Bumala A**	13(3·5)
	**Busia**	82(22.1)
	**Khunyangu**	90(24·3)
	**Kocholia**	30(8·1)
	**Malaba**	3(0·8)
	**Matayos**	7(1·9)
	**Mukhobola**	92(24·8)
	**Port Victoria**	50(13·5)
	Total	371(100)
**Clinic**	**ANC**^**a**^	326(60·8)
	**Postnatal clinic**	187(34·9)
	**MCH**^**b**^	134(25)
	**PMTCT**^**c**^	22(4·1)
	**CCC**^**d**^	1(0·2)

^a^antenatal clinic

^b^maternal and child health

^c^prevention of mother-to-child transmission of HIV

^d^comprehensive care clinic

Table 3 shows that 60.8% of the mothers were counselled on young infant feeding at ANC clinic and that only 4.1% were counselled at PMTCT clinics

### Young infant feeding counseling

There were 536 responses which were not mutually exclusive to the question on when mothers were counseled on young infant feeding. The mothers were counselled at more than one clinic thus resulting into more responses compared to the total number of mothers interviewed. Three hundred and twenty six (61%), 187 (35%), 134 (25%), 22 (4%) and 1 (0·2%) mothers were counselled at antenatal, postnatal, maternal and child health (MCH), PMTCT and comprehensive care clinics (CCC) respectively (Table 3). Six nutritionists (Table 2) took care of mothers at 10 health facilities on a rotary basis. In absence of a nutritionist, counseling of mothers was done by other cadres of staff offering health care services. Health care workers spent no time on other aspects of young infant feeding in the context of maternal HIV infection as per the counseling flow chart derived from HIV and infant feeding counseling tools based on United Nations policies and guidelines. The mean nutritional counseling score was 23·7% (range 6·7–40) which was poor. Quantitative data indicated that a small number of mothers 22 (4·1%) were counseled at PMTCT clinics. No general examination of the infants was done by the clinical officers to pick vital signs such as anemia. Clinical officers spent a maximum of 5 min with the mothers during consultation. They emphasized ART adherence to PMTCT of HIV; the rest was done by mentor mothers and nutritionists. Mothers were referred to the nutritionist only when they were found to be under-weight, growth of the infants was faltering or when they were due for initiation of complementary feeds at the age of 6 months.

Three hundred and forty-four (99.1%) of the mothers chose EBF in order to PMTCT of HIV. There was a significant difference in choice of young infant feeding to PMTCT of HIV rather than due to inadequate breast milk (*P* value < 0·001). Mothers with conditions such as cracked nipples and breast infections such as mastitis and breast abscess did not EBF. The main reason for EBF was understood by 347 (99%) of the mothers who EBF (Table 4). Difference in practice of EBF among those who comprehended the reasons why they had to EBF compared to those who did not was statistically significant (Fisher’s Exact Test *p* value = 0·019).

**Table 4:** Reasons for Choice of Young Infant Feeding, Comprehension & Practice of Exclusive Breastfeeding

		**Exclusive breastfeeding**		**Total**	***P* value**
		Yes (%)	No (%)		
**Reason**	**Inadequate breast milk**	3 (0·9)	8 (57·1)	11(3)	< 0·001
	**PMTCT**^**a**^	344 (99·1)	6 (42·9)	350(97)	
	**Total**	347	14	361	
**Comprehension of exclusive breastfeeding**	Yes	347 (99·4)	2 (9)	367 (98·9)	0·019
	No	2 (0·6)	20 (91)	4 (1·1)	
	Total	349 (100)	22 (100)	371 (100)	

^a^Prevention of Mother-to-child-transmission of HIV

Table 4 shows that mothers who comprehended EBF chose to practice EBF. Only 3 who perceived that breast milk was inadequate EBF

### Maternal perspective on exclusive breastfeeding

One hundred and seventy one (46%) of the mothers felt that health care workers had more influence on the decision of the mothers to initiate and sustain EBF. This was to PMTCT of HIV infection to the infants. Health workers also provided nutritional supplementation in form of CSB. One hundred and ninety-two (52%) and 8 (2%) of the mothers felt that culture to mix feed as early as possible and breast disease influence the decision of whether to EBF or not respectively. However, ninety-five percent of the mothers who mentioned cultural practice as a factor that would negate EBF practiced EBF.

The following themes emerged from the FGDs:

- HIV-infected mothers EBF to prevent infecting their infants with HIV
- EBF did not satisfy infants.
- Inadequate food leads to inadequate breast milk
- Inadequate breast milk leads to mixed feeding
- Nutritional supplementation with corn soy blend supported EBF

The themes were demonstrated by the following quotes:

‘’ We, with HIV infection have no choice but to exclusively breastfeed. Otherwise, our babies will get HIV. Mothers without HIV do not exclusively breastfeed their babies for 6 months. They start other foods by two weeks to 3 months. If I was to be HIV negative I would prefer to breastfeed exclusively for 3 months then start introducing porridge and other foods. Being HIV infected, I only fed my baby on breast milk without adding anything for 6 months due to fear of infecting my baby. Mixed feeding ensures that the baby is satisfied and will not cry all the time. We believe that when babies cry they are hungry. They have to be given other foods like porridge to be fat and healthy’’

(FGD – 36-year old mother)

Another mother said;

‘’When a mother does not have enough food, she decides to introduce other foods to the baby before 6 months due to inadequate breast milk. Whenever I felt hungry I just made corn soy blend porridge and fed on it’’.

(31-year old Mother).

When asked what factors in the community influence feeding infants of HIV-infected mothers, a mother responded:

‘’Health status, income, availability of food and family support. If HIV positive I have been advised to exclusively breastfeed.’’

(37-year old Peasant Farmer).

### Health care worker’s perspective on exclusive breastfeeding

From in-depth interviews with the health care workers the following themes emerged:

- Most HIV-infected mothers are practicing EBF to PMTCT
- Health education by health workers and mentor mothers’ live case demonstrations promoted EBF
- Corn soy blend nutritional supplementation supports EBF
- Teenage motherhood and having to work negatively influenced EBF

These themes are supported by the following quote from an interview with a health care worker who was asked to estimate the proportion of mothers who are able to EBF.

“Approximately 90% of the mothers at our facility are exclusively breastfeeding. This is because of health education that we offer soon after delivery on the importance of EBF and how it should be done. I mean proper latching of the baby onto the breast. Community health workers in conjunction with community health volunteers (one per village) are mobilized to talk to the mothers. They identify those with health problems and refer them to this facility. They also report to us that most of our mothers are actually exclusively breastfeeding.”

(Interview – 24-year old Kenya Registered Community Health Nurse).

When asked what contributed to the practice of EBF among HIV infected mothers, an excerpt of an interview of a PMTCT nurse revealed the following:

“Mothers have been able to EBF due to:

- Assurance by clinicians and mentor mothers of reduced risk of MTCT of HIV
- Adherence to comprehensive care clinic services as offered by USAID-AMPATH Plus project
- Supply of monthly 9 kg rations of corn soy blend flour to the mothers soon after enrolment at PMTCT during pregnancy to the end of breastfeeding. This has been supplied by World Food Program.

During health education talks, pregnant mothers are shown healthy babies who have exclusively breastfed and tested negative for HIV looking healthy. We tell them ‘’you see this one was exclusively breastfed for the first 6 months. See how healthy the baby looks.’’ This makes them opt for exclusive breast feeding. Usually we do not give this information once. We continue teaching them during return visits to remind them about the importance of exclusive breastfeeding and how it can prevent our babies from getting infected with HIV. Every morning between 8·00 - 8·30 am there are mentor mothers on the ministry of health side who deliver the same information.”

(36-year old PMTCT nurse)

The culture of avoidance of breastfeeding to preserve the firmness of breasts among young mothers was identified by the health care workers as one of the factors negating the practice of EBF as indicated by the following quote:

‘Teenage pregnancy is a threat to exclusive breast feeding. These young mothers deliver and go back to school leaving their babies with grandparents who feed the babies on porridge till the mother goes back home. They do not express breast milk because they fear their breasts will fall’’. HIV positive mothers in a state of denial and defaulters do not exclusively breastfeed. Once you tell them to choose between formula and exclusive breastfeeding…I do not know if this is due to illiteracy. They choose mix feeding instead. Maybe they are ignorant. Low level of education makes them unaware of the importance of exclusive breastfeeding. Low socio-economic status reduces exclusive breastfeeding. Most of these mothers are jobless housewives. They focus on looking for money and other means to take care of the family forgetting about exclusive breastfeeding. They start weaning their babies as early as 2 months of age. There is need to:

- Create employment for the mothers in order to generate income
- Health education of teenagers in schools to avoid early pregnancy
- Train health care workers on importance of talking to mothers. We need to be updated because medicine is a dynamic subject. Most trainings are on malaria but not breastfeeding
- Motivate community health volunteers by regular payment for the services they are offering the mothers.”

(24 year old KRCHN)

Due to shortage of staff, healthcare workers may not find time to breastfeed their babies as they provide health care services. One said:

‘’ Working mothers especially health care workers have a problem with exclusive breastfeeding. When they give birth they are transferred far away from home so that they don’t be a nuisance having to rash home to breastfeed. Keeping them far away from home ensures that they work full time. In fact one day our landlord observed that children of working mothers on our plot were undernourished compared to naked children of unemployed mothers. It is very embarrassing because we preach exclusive breastfeeding but practice the opposite. Expressing breast milk is an alternative but mothers working far away from home have no time to express breast milk’’.

(54 year-old nurse)

### Promotion and support of exclusive breastfeeding

It was observed that health care services by trained nurses were complemented by mentor mothers who promoted and supported EBF of HIV-exposed infants by sharing their experiences and offering live case demonstrations (LCDs) of their successfully EBF one and a half year old healthy and HIV-uninfected children. During health education talks by nurses and mentor mothers, HIV-infected mothers were informed that EBF would prevent transmitting HIV infection to the infants if they adhered to antiretroviral treatment. Three hundred and forty-nine (94%) of the mothers practiced EBF for 6 months.

### Maternal and infant morbidity

Three hundred and six (83%) of the infants suffered no morbidity, 19 (5%), 14 (4%), and 10 (3%) suffered from malaria, upper respiratory tract infection (URTI) and gastroenteritis respectively.

The following FGD quote corroborates this finding:

‘’ I have 3 children and the first two I practiced mixed feeding and they used to be sickly. This one has never fallen ill.’’

(FGD – 27-year old mother)

Three hundred and thirty one (92%) of the mothers suffered no health problems, 8 (2%) had breast conditions while 7 (2%) suffered recurrent infections. Thirteen (4%) suffered weight loss, hunger, weakness and dizziness due to inadequate food yet they had to EBF.

One mother said:

‘’ My CD4 count was doing very well. But when I started breastfeeding…It started going down then I started falling sick every now and then.’’

(33 year old mother)

## Discussion

The findings of our study demonstrate that health care workers at PMTCT of HIV clinics in Busia County, western Kenya were instrumental in achieving 94% rate of exclusive breastfeeding. These were mainly females who cut across most cadres of staff required to offer comprehensive care. Being in the reproductive age bracket, the health workers were in a better position to articulate factors influencing EBF. Health care services to PMTCT were complemented by mentor mothers and community health volunteers designed to enhance adherence to antiretroviral treatment, promotion and support EBF of HIV-exposed infants. Nutritional supplementation aided in sustaining the practice of EBF for 6 months.

Prevention of mother-to-child transmission of HIV through provision of ART to the mother and breastfeeding infant is one of the main methods geared towards eradication of HIV pandemic in the future generations. This can only be achieved if breastfeeding mothers adhere to ART to ovoid development of resistance to the virus, high viral load and transmission of the virus to the infant. In our study, involvement of mentor mothers in provision of health care to breastfeeding mothers improved adherence to ARVs. Similarly provider-patient communication has been linked to patient satisfaction, adherence to medical instructions, and health outcomes [29]

Young infant feeding counseling by health care workers is an essential prerequisite for choice of infant feeding in the context of maternal HIV infection. In our study mothers were informed and understood the meaning of EBF. They made informed choice to EBF following counseling on the need to adhere to antiretroviral treatment as they EBF in order to PMTCT of HIV. This promoted the practice of EBF in this population. Similarly a realist review in sub-Saharan Africa analyzing the use of feeding counselling to increase EBF by HIV-positive women revealed that EBF occurred when a woman was motivated regarding motherhood, had correct learning and understanding about infant feeding practices through counselling [30]. Among HIV-infected mothers living with HIV in Kiambu County, Kenya, EBF rate was 71.4%, mixed feeding 18.2% and replacement feeding 10.4%. Age and infant feeding practice knowledge were determinants of EBF. Poor infant feeding practice knowledge led to non-adherence to EBF [31]. In our study most mothers received counseling on young infant feeding at ANC, postnatal and MCH rather than PMTCT clinics. These data emphasize the need for mothers to attend ANC and MCH clinics where they receive health education by health care workers. Similarly, systematic review and meta-analysis of 32 international studies revealed that mothers who attended ANC were likely to practice EBF than their counterparts [32].

The criteria for selection of a mentor mother were: HIV-infected mothers with a healthy HIV-uninfected child at 18 months of age, strict adherence to antiretroviral therapy, successful EBF for 6 months, and good communication skills. They served as peer counselors to the mothers which enhanced ARVs adherence and practice of EBF. Mentor mothers also offered live case demonstrations of their HIV-uninfected 18-month old children who had been EBF for 6 months against the society norm of early introduction of other foods. Similarly in Nairobi, Kenya, some women were able to EBF regardless of opposing social norms due to health education with peer counselors and male partner support [33] Among Latinos, a systematic review designed to assess the impact of peer education/counseling on nutrition and health outcomes revealed that peer education has a positive influence on breastfeeding and the general nutrition knowledge [34]. In England, breastfeeding support including that delivered by support workers was found to have important role in preventing cessation of breastfeeding in the first weeks of life [35]. In South Africa, augmenting basic PMTCT services with mentor mothers and a culturally adapted cognitive behavioral intervention was effective in conveying information and in improving the emotional outlook and hopefulness of HIV positive pregnant women [36]. Education by demonstration makes the mothers adopt and sustain the practice more easily because they are given the opportunity to observe the results of changed health seeking behavior. Similarly, an internet based survey of clinicians has demonstrated that observing live case demonstrations (LCDs) is more valuable than watching a recorded video [37].

Most mothers, therefore, chose to EBF their infants in order to PMTCT of HIV. The mothers strictly followed health workers’ advice thus making the health worker an important factor in choice of young infant feeding in the context of maternal HIV infection. Similarly in Zambia the meanings attached to exclusive breastfeeding influenced their understanding of mixed feeding in the context of PMTCT of HIV [38]. In our study, all mothers with breast disease were advised not to breastfeed because breast disease has been associated with increased MTCT of HIV [39].

The fear of MTCT of HIV if they mixed fed their infants instilled into the mothers by health workers through health education contributed to the high rate of EBF in this population. This is contrary to a meta-analysis that tested the major theoretical assumptions about behavior change by examining the outcomes and mediating mechanisms of different preventive strategies in a sample of 354 HIV-prevention interventions and 99 control groups spanning the past 17 years [40]. This review concluded that the most effective interventions were those that contained attitudinal arguments, educational information, behavioral skills arguments and behavioral skills training while the least effective ones were those that attempted to induce fear of HIV. This is unlike our study where besides inducing fear to change infant feeding practice, mothers were advised to EBF following an assessment of their socioeconomic status. They were also educated on the advantages of EBF such as prevention of other infections including diarrhea and respiratory tract infections besides PMTCT of HIV. Another systematic review of behavioral counseling interventions which sought to establish whether such interventions addressing individual behaviors improve health outcomes found no simple empirically validated model which captured the broad range of intervention components across risky behaviors except the five A’s construct – assess, advise, agree, assist and arrange [41].

Nutritional counseling by health care workers was, however, rated poor due to emphasis on adherence to ART only by clinical officers who were the majority, leaving the rest to be done by nutritionists and mentor mothers. This could be due to the large number of patients seen by the clinical officers at PMTCT clinics. Alternatively poor knowledge of young infant feeding could explain the poor score as was seen in Ibadan where the knowledge of primary health care workers on infant and young child feeding was poor prior to training [42]. In Ghana health talks that were shared with caregivers focused mainly on the importance of attending child welfare clinics and vaccination and rarely included any teaching materials [43]. In Zambia nurses interviewed reported to be overwhelmed by the high demand for counselling services resulting into compromise on quality [44].

These data demonstrates the importance of ensuring that the information delivered to the mothers during health education is precise. This emphasizes the need for up-to date knowledge of health workers on young infant feeding counseling in this context. USAID-AMPATH Plus project regularly updated the knowledge of health care workers on young infant feeding through regular in-service training. This is unlike Papua New Guinea where key health workers are not aware of up-to date information on infant feeding in the context of HIV [45].

Health care workers’ provision of 9 kg monthly rations of corn soy blend supplied by World Food Program supported the mothers to produce enough breast milk required to sustain EBF for 6 months. These made the mothers embrace the practice of EBF contrary to the cultural norm of early introduction of other foods before infants attain the age of 6 months and resulted in reduced under-five morbidity in this population. Most infants did not suffer morbidity from malaria, upper respiratory tract infection and gastroenteritis respectively. Likewise, most mothers suffered no health problems.

These data demonstrates that following acceptable adoption of the practice of EBF, it is crucial to support the mothers in order to sustain the practice. USAID-AMPATH Plus project addressed this through integrated health care approach that took care of the mothers’ health, nutritional and psycho-social needs through outreach services, psychosocial support and regular training of relevant staff. The project conducts training needs assessment during monitoring and evaluation of its activities. This identifies the various training aspects required to deliver the service to meet the client needs. Indeed EBF on a population basis has been shown to be feasible with adequate support and training of health care professionals [46]. The main threats to EBF were teenage motherhood, low maternal education and having to work during the period of EBF.

### Study limitations

A cross sectional study design could not ascertain whether the mothers actually practiced EBF or not. The results cannot be generalized because subjects were sampled purposively rather than randomly. Mothers’ responses to questions could have been biased due to the hospital setting of the study which could have prompted them to give expected responses.

## Conclusion

EBF among HIV-infected mothers in Busia County of Kenya was high. Health education and counselling by health care workers emphasizing ART adherence to PMTCT of HIV during EBF influenced HIV-infected mothers’ decision to choose and practice EBF. Corn soy blend nutritional supplementation and mentor mothers’ peer counseling using live case demonstrations of HIV-uninfected EBF children sustained the practice of EBF for 6 months. The number of health care workers at PMTCT clinics were inadequate to comprehensively counsel the mothers on young infant feeding and offer clinical care. The main threats to EBF were teenage motherhood, low maternal education and having to work during EBF. Future strategies to support and sustain EBF need to focus on nutritional counseling using LCDs and nutritional supplementation.

## Data Availability

Datasets used and/or analyzed during the current study are available from the corresponding author on reasonable request.

## Abbreviations

AMPATH: Academic Model Providing Access to Health Care
ANC: Antenatal care
ART: Antiretroviral therapy
ARVs: Antiretroviral drugs
EBF: Exclusive breastfeeding
FANTA: Food and Nutrition Technical Assistance
FGDs: Focus Group Discussions
HIV: Human Immunodeficiency Virus
KDHS: Kenya Demographic and Health Survey
KECN: Kenya Enrolled Community Nurse
KRCHN: Kenya Registered Community Health Nurse
MCH: Maternal and child health clinics
MTCT: Mother-to-child transmission
PMTCT: Prevention of mother-to-child transmission
UNAIDS: United Nations Program on HIV and AIDS
UNFPA: United Nations Population Fund
UNICEF: United Nations Children’s Fund
USAID: United States Urgency for International Development
WHO: World Health Organization
